# Predictive Value of Plasma MicroRNA-216a/b in the Diagnosis of Esophageal Squamous Cell Carcinoma

**DOI:** 10.1155/2016/1857067

**Published:** 2016-02-18

**Authors:** Shuling Dong, Huiqing Yin, Cuicui Dong, Kaiyan Sun, Pin Lv, Weiwei Meng, Liang Ming, Fucheng He

**Affiliations:** ^1^Department of Medical Laboratory, The First Affiliated Hospital of Zhengzhou University, Zhengzhou, Henan 450052, China; ^2^Key Medical Laboratory of Henan Province, Zhengzhou, Henan 450052, China; ^3^Department of Medical Laboratory, The Second Affiliated Hospital of Zhengzhou University, Zhengzhou, Henan 450052, China

## Abstract

Esophageal squamous cell carcinoma (ESCC) is a common human malignancy with poor survival, which was usually diagnosed at an advanced stage. MicroRNAs (miRNAs), a class of single stranded noncoding RNAs with only 17–25 ribonucleotides, were demonstrated to play an important role in lots of cancers. In the recent years, increasing evidence revealed that circulating miRNAs exhibited great potential in the diagnosis of various types of cancers. The present study was designed to evaluate the diagnostic value of plasma miRNA-216a/b for ESCC. Our results showed that the expression level of plasma miRNA-216a/b was significantly lower in ESCC patients compared with that of healthy controls. The receiver operating characteristic (ROC) curve analysis yielded an area under the ROC curve (AUC) value of 0.877 [95% CI (confidence interval): 0.818–0.922] for miRNA-216a and 0.756 (95% CI: 0.685–0.819) for miRNA-216b. Clinical data indicated that plasma miRNA-216a/b were inversely correlated with lymph node metastasis and TNM stage. Additionally, the plasma miRNA-216b expression level was significantly upregulated in postoperative samples compared to preoperative samples. Our study, for the first time, demonstrated that plasma miRNA-216a/b might serve as potential biomarkers for the diagnosis of ESCC and dysregulation of miRNA-216a/b might be involved in the progression of ESCC.

## 1. Introduction

Esophageal carcinoma (EC), one of the most common human malignancies, ranks eighth in incidence and sixth in cancer death worldwide [1]. There are two major histological subtypes of EC: esophageal squamous cell carcinoma (ESCC) and esophageal adenocarcinoma (EAC) [1]. Despite the advances in medical and surgical treatments, most EC patients are diagnosed at an advanced stage because of the few early symptoms, thus causing a poor prognosis with an overall five-year survival ranging from 15% to 25% [2]. Therefore, the early diagnosis is essential for improving the prognosis of EC patients. Conventional serum tumor biomarkers, such as squamous cell carcinoma antigen (SCC-Ag) and carcinoembryonic antigen (CEA), had important value for the early diagnosis of EC [3]. However, the insufficient sensitivity and specificity badly restrict their clinical application value. Hence, novel biomarkers with sufficient sensitivity and specificity are urgently needed to be discovered for the early diagnosis of EC.

MicroRNAs (miRNAs) are a class of single stranded noncoding RNAs with only 17–25 ribonucleotides [4]. They can inhibit translation or promote degradation of mRNAs by imperfect base paring with the 3′ untranslated regions (3′-UTRs) of the target mRNAs, thus regulating almost 30%–60% of human genes expression [5, 6]. Since the first discovery in* Caenorhabditis elegans* in 1993 [7], miRNAs have been demonstrated to be involved in many biological processes including cell differentiation, proliferation, apoptosis, and growth control [8] and presented great potential roles in the development and progression of various types of human diseases [9]. In 2008, Lawrie et al. [10] found that miRNAs could be clearly detectable in serum samples and that the dysregulation of miRNAs was correlated with the diagnosis and prognosis of diffuse large B-cell lymphoma patients, which initiated the investigations on the role of circulating miRNAs in the diagnosis of human diseases. Accumulating studies have demonstrated the remarkably stable existence of miRNAs in the plasma or serum [11, 12], thus heightening the feasibility of circulating miRNAs serving as novel and reliable diagnostic biomarkers. A considerable number of studies reported that plasma or serum miRNAs could serve as potential biomarkers for the detection of ESCC [13–16].

Recently, miRNA-216a/b, as two members of miRNA-216 family, have been demonstrated to be dysregulated in several types of human cancers. miRNA-216a was found to be downregulated in non-small-cell lung cancer (NSCLC) [17] and oral squamous cell carcinoma (OSCC) [18], while it was found to be upregulated in hepatocellular carcinoma (HCC) [19, 20]. miRNA-216b was downregulated in nasopharyngeal carcinoma (NPC) [21] and HCC [22]. What is more, the diagnostic value of miRNA-216a/b was also demonstrated in several diseases including acute pancreatitis [23, 24], pancreatic cancer (PCA) [25], and HCC [22]. Despite these findings, the role of miRNA-216a/b in ESCC has never been reported previously. The present study was designed to examine the expression of plasma miRNA-216a/b in ESCC patients and evaluate their diagnostic value, hoping to provide some valuable information in the early diagnosis of ESCC.

## 2. Materials and Methods

### 2.1. Patients and Samples

A total of 120 consecutive patients of ESCC at the First Affiliated Hospital of Zhengzhou University who were newly diagnosed and previously untreated (including surgery, chemotherapy, and radiotherapy) were recruited from April 2014 to June 2015. All patients' clinicopathological characteristics including age, gender, smoking, alcohol use, tumor location, histologic grade, T stage, lymph node metastasis, and TNM stage were presented in Table 1. Tumors were staged according to the TNM staging system of the Union for International Cancer Control (UICC). Histologic grade was assessed according to World Health Organization (WHO) criteria. As controls, 51 individuals matching age and gender of the ESCC patients, who sought a routine health check-up and did not have any esophageal diseases or other cancerous diseases, were recruited at the same period. Fasting venous blood samples (2 mL) from each participant were collected in tubes containing EDTA-K_2_ with informed consent and agreement, according to protocols approved by the Ethics Committee of the First Affiliated Hospital of Zhengzhou University. Postoperative venous blood samples from 21 patients were collected 1 month after esophagectomy. Immediately after collection, the blood samples were centrifuged at 3000 ×g for 10 min at 4°C; then the supernatant was isolated and centrifuged at 12,000 ×g for 10 min at 4°C to prevent contamination by cellular nucleic acids. Plasma samples were then stored in aliquots at −80°C until further processing.

### 2.2. RNA Extraction, Reverse Transcription, and Quantitative Real-Time PCR (qRT–PCR)

Total RNA was extracted from each plasma sample using Trizol reagent BD (MRC, TB-126, Cincinnati, OH, USA) and then reverse-transcribed using a PrimeScript*™* RT Reagent Kit (TaKaRa, DRR047A, Tokyo, Japan) according to the manufacturer's instructions. After that, qRT-PCR was performed to quantify the expression levels of plasma miRNAs using SYBR^OeR^ Premix Ex Taq*™* II (TaKaRa, DRR820S, Tokyo, Japan). The endogenous miRNA-16 was used as reference gene, which was demonstrated to be feasible in several studies [13, 26, 27]. Each reaction was performed in triplicate. Data analysis was performed with 7500 Fast System SDS software version 1.4.0.25 (Applied Biosystems, Foster City, CA, USA). miRNAs relative expression levels were calculated by the equation of 2^−ΔCt^  (ΔCt = Ct_target_ − Ct_miRNA-16_).

### 2.3. Statistical Analysis

SPSS Statistics software version 17.0 (SPSS Inc., USA) was used for the statistical analysis. All continuous data were presented as mean ± SD and categorical variables were presented as counts and percentage. The unpaired Student's *t*-test or Mann-Whitney *U* test was performed to compare the difference of miRNAs expression levels between two groups. The paired *t*-test was utilized to compare the difference of miRNAs expression levels between paired groups. The one-way ANOVA or Kruskal-Wallis test was performed to compare the difference of miRNAs expression levels among three or more groups. Receiver operating characteristic (ROC) curve and the area under the ROC curve (AUC) were used to evaluate the diagnostic power of plasma miRNAs for ESCC. All tests were two-sided, and the significance level was set at *P* < 0.05.

## 3. Results

### 3.1. Downregulation of Plasma miRNA-216a/b in ESCC Patients

**Figure 1:**
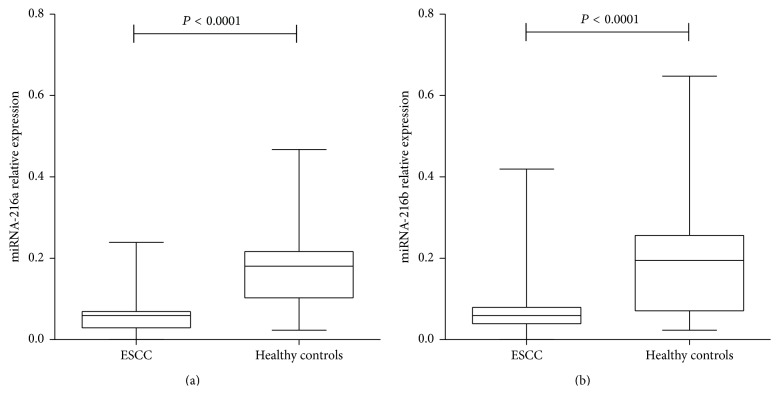
Difference of plasma miRNA-216a/b expression level between ESCC patients and healthy controls. The expression level of plasma miRNA-216a (a) and miRNA-216b (b) in ESCC patients was significantly lower than that of healthy controls.

The qRT-PCR method was performed to detect the expression level of plasma miRNA-216a/b in 120 ESCC patients and 51 healthy controls. Then, the difference was analyzed. As shown in Figure 1, the expression level of miRNA-216a/b was significantly downregulated in ESCC patients compared with that of healthy controls (0.068 ± 0.052 versus 0.179 ± 0.098, *P* < 0.0001; 0.091 ± 0.087 versus 0.199 ± 0.161, *P* < 0.0001) (Figures 1(a) and 1(b)).

### 3.2. Influence of Operation on the Expression Level of Plasma miRNA-216a/b in ESCC Patients

**Figure 2:**
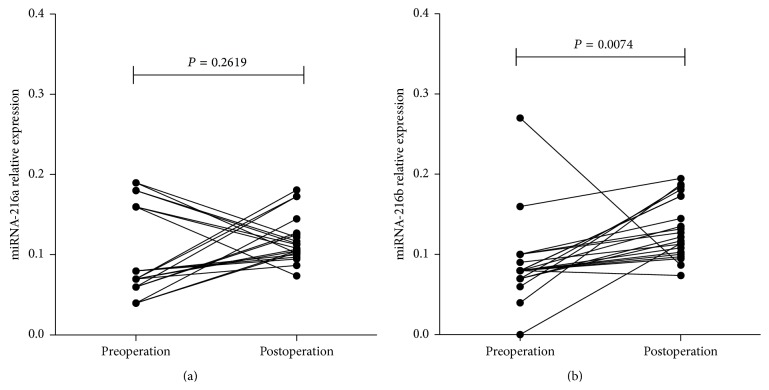
Difference of plasma miRNA-216a/b expression level between preoperative and postoperative samples. The expression level of plasma miRNA-216b was significantly upregulated in postoperative samples compared to that of preoperative samples (b), while no significant difference was found in miRNA-216a (a).

We next examined the value of plasma miRNA-216a/b in the evaluation of surgical efficacy. The postoperative venous blood samples from 21 patients were collected 1 month after esophagectomy. Paired *t*-test was performed to analyze the difference of plasma miRNA-216a/b expression level between preoperative and postoperative samples. We found that the expression level of plasma miRNA-216b in postoperative samples was significantly upregulated compared with that of preoperative samples (0.132 ± 0.037 versus 0.088 ± 0.051, *P* = 0.0074) (Figure 2(b)). Although the expression level of plasma miRNA-216a was also upregulated after esophagectomy, the result was insignificant (*P* = 0.2619) (Figure 2(a)).

### 3.3. Correlation of Plasma miRNA-216a/b with the Clinicopathological Characteristics of ESCC Patients

**Figure 3:**
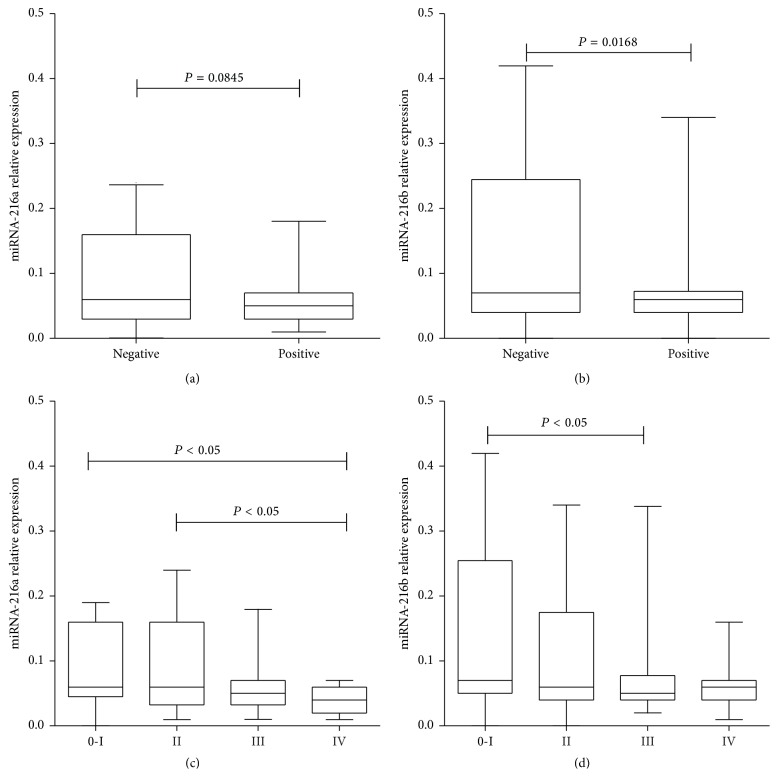
Correlation of plasma miRNA-216a/b with lymph node metastasis and TNM stage. Patients with lymph node metastasis exhibited significantly lower plasma miRNA-216b expression level than those without lymph node metastasis (b), while no significant difference was found in miRNA-216a (a). Patients with TNM IV exhibited significantly lower plasma miRNA-216a expression level than those with TNM 0-I or II (c). Patients with TNM III exhibited significantly lower plasma miRNA-216b expression level than those with TNM 0-I (d).

We analyzed the correlation of plasma miRNA-216a/b expression level with the clinicopathological characteristics of 120 ESCC patients. Results revealed that patients with lymph node metastasis showed significantly lower miRNA-216b expression level than patients without lymph node metastasis (0.068 ± 0.054 versus 0.134 ± 0.117, *P* = 0.0168) (Figure 3(b)), while the miRNA-216a expression level showed no significant difference (*P* = 0.0845) (Figure 3(a)); patients with TNM IV showed significantly lower miRNA-216a expression level than patients with TNM 0-I or TNM II (0.039 ± 0.022 versus 0.088 ± 0.062, *P* < 0.05; 0.039 ± 0.022 versus 0.086 ± 0.065, *P* < 0.05) (Figure 3(c)); and patients with TNM III showed significantly lower miRNA-216b expression level than patients with TNM 0-I (0.072 ± 0.067 versus 0.134 ± 0.119, *P* < 0.05) (Figure 3(d)). There was no significant difference of miRNA-216a/b among other clinicopathological characteristics (Table 1).

### 3.4. The Diagnostic Value of Plasma miRNA-216a/b for ESCC

**Figure 4:**
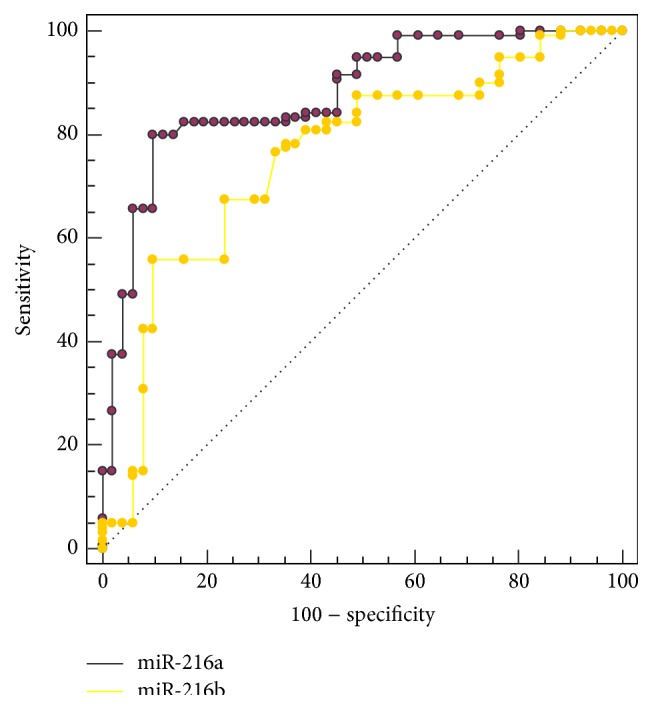
ROC curve analysis for plasma miRNA-216a/b in the diagnosis of ESCC.

ROC curve analysis was performed to evaluate the diagnostic value of plasma miRNA-216a/b for ESCC. We found that plasma miRNA-216a/b could differentiate ESCC patients from healthy controls, with an AUC of 0.877 (95% CI: 0.818–0.922) for miRNA-216a and 0.756 (95% CI: 0.685–0.819) for miRNA-216b, respectively. Plasma miRNA-216a was superior to miRNA-216b in the diagnosis of ESCC (*z* = 3.141; *P* = 0.0017) (Figure 4). At the cut-off value of 0.070, the sensitivity was 80.0% and the specificity was 90.2% for miRNA-216a; and at the cut-off value of 0.060, the sensitivity was 55.8% and the specificity was 90.2% for miRNA-216b.

## 4. Discussion

It is generally considered that early diagnosis is crucial to the survival and prognosis of various types of cancers. Despite the great contribution of conventional biomarkers to the diagnosis of human cancers, the insufficient sensitivity and specificity discount their diagnostic value. Therefore, novel and reliable biomarkers are urgently needed to be discovered for the early diagnosis of cancers.

By packing into exosomal or bounding to Argonaute2 (Ago2) protein, miRNAs can exist stably in the blood stream [27, 28]. Previous reports demonstrated that plasma miRNAs were not derived from blood cells and their contents were similar to those in the original tumors [27, 29]. Moreover, the expression level of circulating miRNAs was reproducible and consistent among individuals [27, 30]. All the characteristics mentioned above strongly supported the feasibility to use circulating miRNAs as potential diagnostic biomarkers for human cancers and other diseases. In fact, accumulating evidence demonstrated that circulating miRNAs could serve as potential diagnostic biomarkers for a wide range of human cancers [30].

miRNA-216a/b, two members of miRNA-216 family, have been demonstrated to be dysregulated in several types of human cancers. Deng et al. [21] found that miRNA-216b was downregulated in NPC cell lines and specimens and played a tumor suppressive role in NPC by targeting KRAS. In cooperation with other three miRNAs, miRNA-216b could induce cellular senescence through the p53-p21^Cip1/WAF1^ pathway by protein kinase CKII downregulation-mediated ROS production in human colorectal cancer cells [31]. In HCC, miRNA-216b could function as a tumor suppressor by targeting IGF2BP2 and subsequently suppressing the downstream IGF2 [22]. Decreased expression of miRNA-216a was found in NSCLC patients' specimens and by directly targeting eIF4B and ZEB1, miRNA-216a could inhibit NSCLC cell growth and metastasis [17]. Similar roles and mechanism of miRNA-216a were also found in OSCC [18]. In contrast with the tumor suppressive role of miRNA-216a/b mentioned above, the following studies held the opposite opinion. miRNA-216a was significantly upregulated and contributed to early hepatocarcinogenesis through suppressing the gene expression of tumor suppressor in lung cancer-1 (TSLC1) [19], which was in accordance with Xia et al.'s report that miRN-216a could induce epithelial-mesenchymal transition by targeting PTEN and SMAD7, thus contributing to hepatocarcinogenesis and recurrence [20]. Additionally, miRNA-216a/b might influence the therapeutic effect of different treatments, thus providing candidate therapeutic methods for different cancers. For example, overexpression of miRNA-216a could induce resistance of sorafenib in HCC cells [20]. In other reports, however, it could enhance the sensitivity of NSCLC cells to CDDP treatment [17] and enhance the radiosensitivity of pancreatic cancer cells by inhibiting beclin-1-mediated autophagy [32]. In the recent years, the diagnostic value of miRNA-216a/b has drawn more and more attention. Plasma miRNA-216a/b were found to be considerably upregulated in the model of acute pancreatitis and were more specific than amylase and lipase in the detection of acute pancreatitis [23, 24], providing novel biomarkers for pancreatic injury. Decreased expression of miRNA-216a in feces might be used as potential screening biomarkers for pancreatic cancer [25]. In HCC, plasma miRNA-216b was revealed to be significantly downregulated compared with healthy volunteers [22].

Despite the meaningful value of miRNA-216a/b in the diseases mentioned above, there has been no research on the role of miRNA-216a/b in ESCC up to now. In the present study, we discovered, for the first time, that patients with ESCC had significantly lower plasma miRNA-216a/b expression level compared with healthy controls. ROC curve analysis showed that plasma miRNA-216a/b exhibited satisfactory diagnostic value for ESCC, with AUC of 0.877 (95% CI: 0.818–0.922) for miRNA-216a and 0.756 (95% CI: 0.685–0.819) for miRNA-216b, which were higher than conventional biomarkers such as SCC-Ag (AUC: 0.665) and CEA (AUC: 0.549) reported in other studies [13, 16]. Our results revealed that plasma miRNA-216a/b might serve as potential diagnostic biomarkers for ESCC. Further analysis revealed that plasma miRNA-216a/b were inversely correlated with lymph node metastasis and TNM stage, indicating that miRNA-216a/b might be involved in the progression of ESCC. Therefore, it seems important and valuable to investigate the exact mechanism of miRNA-216a/b in the development and progression of ESCC. What is more, we found that the expression level of plasma miRNA-216b after esophagectomy was significantly upregulated, thus providing a useful insight into the application of plasma miRNA-216b in the evaluation of therapeutic effect. Indeed, previous studies have demonstrated the value of circulating miRNAs in the evaluation of different therapeutic methods [13, 33–35]. As a homologous miRNA of miRNA-216b, theoretically, miRNA-216a is expected to exhibit similar expression after esophagectomy. However, the data in the present study show no significant change. The possible reason is due to heterogeneous distribution of miRNA-216a in the study subjects after esophagectomy. Among the 21 patients who underwent esophagectomy, the number (proportion) of patients with upregulated miRNA-216b expression after esophagectomy was 19 (90.5%), while the number (proportion) was only 14 (66.7%) for miRNA-216a. In our study, the expression level of plasma miRNA-216a in postoperative samples was indeed upregulated compared with that of preoperative samples (0.118 ± 0.028 versus 0.101 ± 0.056), although it was insignificant (*P* = 0.2619). If we expanded the sample size, miRNA-216a would probably exhibit significant upregulation. Therefore, it might be the small sample size that resulted in the inconformity between miRNA-216a and miRNA-216b.

Some limitations of our study must be addressed. Firstly, the sample size in our study was relatively small and only cross-sectional samples were used to compare the difference of plasma miRNA-216a/b between ESCC patients and healthy controls, which might result in bias in the final results. Hence, longitudinal study with larger sample size is needed to confirm the practical value in clinical use. Secondly, miRNA-216a/b seem not to be specific miRNAs in ESCC. Therefore, it might be better to combine conventional biomarkers and/or other miRNAs to obtain a more satisfactory diagnosis for ESCC. Finally, it is a pity that we could not gain enough data to perform survival analysis. Therefore, the prognostic value of plasma miRNA-216a/b was unknown. A longer follow-up survey is definitely needed to evaluate the prognostic significance of plasma miRNA-216a/b in ESCC.

## 5. Conclusions

In conclusion, our study demonstrated that plasma miRNA-216a/b might act as potential diagnostic biomarkers for ESCC. Dysregulation of miRNA-216a/b was correlated with the lymph node metastasis and TNM stage of ESCC patients, thus indicating that they might be involved in the progression of ESCC. In order to have a better understanding of the role of miRNA-216a/b in ESCC, further efforts are recommended to reveal the exact mechanism of miRNA-216a/b in the carcinogenesis of ESCC.

## Figures and Tables

**Table 1:** Clinicopathologicalcharacteristics of patients with ESCC.

Characteristics	*n* (%)	Plasma miRNA-718 expression (mean ± SD)			
		miR-216a	*P* value	miR-216b	*P* value
Age (year)			0.1082		0.2125
≤63	46 (38.3)	0.058 ± 0.048		0.079 ± 0.086	
>63	74 (61.7)	0.074 ± 0.054		0.099 ± 0.087	
Gender			0.5388		0.5912
Male	79 (65.8)	0.070 ± 0.055		0.094 ± 0.087	
Female	41 (34.2)	0.064 ± 0.048		0.085 ± 0.089	
Smoking			0.1342		0.6168
Never	52 (43.3)	0.060 ± 0.047		0.087 ± 0.079	
Ever	68 (56.7)	0.074 ± 0.056		0.095 ± 0.093	
Alcohol use			0.2378		0.5386
Never	40 (33.3)	0.060 ± 0.054		0.098 ± 0.087	
Ever	80 (66.7)	0.072 ± 0.051		0.088 ± 0.087	
Tumor location			0.3136		0.1810
Upper esophagus	11 (9.2)	0.065 ± 0.061		0.106 ± 0.092	
Middle esophagus	70 (58.3)	0.074 ± 0.056		0.100 ± 0.096	
Low esophagus	39 (32.5)	0.058 ± 0.041		0.072 ± 0.066	
Histologic grade			0.8959		0.8308
Well differentiated	33 (27.5)	0.070 ± 0.059		0.109 ± 0.112	
Moderately differentiated	70 (58.3)	0.068 ± 0.052		0.089 ± 0.082	
Poorly differentiated	17 (14.2)	0.063 ± 0.040		0.068 ± 0.030	
T stage			0.3206		0.6600
Tis-T1	36 (30.0)	0.075 ± 0.061		0.115 ± 0.114	
T2	28 (23.3)	0.086 ± 0.065		0.100 ± 0.090	
T3	41 (34.2)	0.056 ± 0.037		0.074 ± 0.065	
T4	15 (12.5)	0.050 ± 0.019		0.067 ± 0.031	
Lymph node metastasis			0.0845		**0.0168**
Negative	42 (35.0)	0.088 ± 0.068		0.134 ± 0.117	
Positive	78 (65.0)	0.057 ± 0.038		0.068 ± 0.054	
TNM stage			**0.0054**		**0.0359**
0-I	29 (24.2)	0.088 ± 0.062		0.134 ± 0.119	
II	28 (23.3)	0.086 ± 0.065		0.101 ± 0.090	
III	40 (33.3)	0.058 ± 0.035		0.072 ± 0.067	
IV	23 (19.2)	0.039 ± 0.022		0.060 ± 0.029	
