# Corrigendum to “Predictive Value of Plasma MicroRNA-216a/b in the Diagnosis of Esophageal Squamous Cell Carcinoma”

**DOI:** 10.1155/2017/3437679

**Published:** 2017-03-12

**Authors:** Shuling Dong, Huiqing Yin, Cuicui Dong, Kaiyan Sun, Pin Lv, Weiwei Meng, Liang Ming, Fucheng He

**Affiliations:** ^1^Department of Medical Laboratory, The First Affiliated Hospital of Zhengzhou University, Zhengzhou, Henan 450052, China; ^2^Key Medical Laboratory of Henan Province, Zhengzhou, Henan 450052, China; ^3^Department of Medical Laboratory, The Second Affiliated Hospital of Zhengzhou University, Zhengzhou, Henan 450052, China

In the article titled “Predictive Value of Plasma MicroRNA-216a/b in the Diagnosis of Esophageal Squamous Cell Carcinoma” [1], there was an error in Table 1. The heading incorrectly referred to miRNA-718 expression instead of miRNA-216a/b. miRNA-718 expression is the subject of a related article, Sun L, Dong S, Dong C, Sun K, Meng W, Lv P, Yin H, Ming L, He F: “Predictive value of plasma miRNA-718 for esophageal squamous cell carcinoma”. Cancer Biomark. 2016;16(2):265-73. doi: 10.3233/CBM-150564. The table should be corrected as follows.

## Figures and Tables

**Table 1:** Clinicopathological characteristics of patients with ESCC.

Characteristics	*n* (%)	Plasma miRNA relative expression level (mean ± SD)			
		miR-216a	*P* value	miR-216b	*P* value
Age (year)			0.1082		0.2125
≤63	46 (38.3)	0.058 ± 0.048		0.079 ± 0.086	
>63	74 (61.7)	0.074 ± 0.054		0.099 ± 0.087	
Gender			0.5388		0.5912
Male	79 (65.8)	0.070 ± 0.055		0.094 ± 0.087	
Female	41 (34.2)	0.064 ± 0.048		0.085 ± 0.089	
Smoking			0.1342		0.6168
Never	52 (43.3)	0.060 ± 0.047		0.087 ± 0.079	
Ever	68 (56.7)	0.074 ± 0.056		0.095 ± 0.093	
Alcohol use			0.2378		0.5386
Never	40 (33.3)	0.060 ± 0.054		0.098 ± 0.087	
Ever	80 (66.7)	0.072 ± 0.051		0.088 ± 0.087	
Tumor location			0.3136		0.1810
Upper esophagus	11 (9.2)	0.065 ± 0.061		0.106 ± 0.092	
Middle esophagus	70 (58.3)	0.074 ± 0.056		0.100 ± 0.096	
Low esophagus	39 (32.5)	0.058 ± 0.041		0.072 ± 0.066	
Histologic grade			0.8959		0.8308
Well differentiated	33 (27.5)	0.070 ± 0.059		0.109 ± 0.112	
Moderate differentiated	70 (58.3)	0.068 ± 0.052		0.089 ± 0.082	
Poor differentiated	17 (14.2)	0.063 ± 0.040		0.068 ± 0.030	
T stage			0.3206		0.6600
Tis-T1	36 (30.0)	0.075 ± 0.061		0.115 ± 0.114	
T2	28 (23.3)	0.086 ± 0.065		0.100 ± 0.090	
T3	41 (34.2)	0.056 ± 0.037		0.074 ± 0.065	
T4	15 (12.5)	0.050 ± 0.019		0.067 ± 0.031	
Lymph node metastasis			0.0845		**0.0168**
Negative	42 (35.0)	0.088 ± 0.068		0.134 ± 0.117	
Positive	78 (65.0)	0.057 ± 0.038		0.068 ± 0.054	
TNM stage			**0.0054**		**0.0359**
0-I	29 (24.2)	0.088 ± 0.062		0.134 ± 0.119	
II	28 (23.3)	0.086 ± 0.065		0.101 ± 0.090	
III	40 (33.3)	0.058 ± 0.035		0.072 ± 0.067	
IV	23 (19.2)	0.039 ± 0.022		0.060 ± 0.029	
